# The Influence of Residual Feed Intake and Cow Age on Beef Cattle Performance, Supplement Intake, Resource Use, and Grazing Behavior on Winter Mixed-Grass Rangelands

**DOI:** 10.3390/ani11061518

**Published:** 2021-05-23

**Authors:** Cory T. Parsons, Julia M. Dafoe, Samuel A. Wyffels, Timothy DelCurto, Darrin L. Boss

**Affiliations:** 1 Northern Agricultural Research Center, Montana State University, Havre, MT 59501, USA; julia.dafoe@montana.edu (J.M.D.); samwyffels@montana.edu (S.A.W.); dboss@montana.edu (D.L.B.); 2 Department of Animal and Range Sciences, Montana State University, Bozeman, MT 59717, USA; timothy.delcurto@montana.edu

**Keywords:** beef cattle, cow age, grazing behavior, residual feed intake (RFI), resource use, supplement intake

## Abstract

**Simple Summary:**

Feed efficiency is becoming an important selection tool in the beef cattle industry. Traditionally, feed efficiency of beef cattle has been expressed as the ratio of feed intake to body weight gained; however, selection for high growth rates inevitably increases the maintenance requirements, feed requirements, and intake of cattle, with subsequent higher feed costs. In contrast, net feed efficiency, or residual feed intake (RFI), is defined as the difference between an animal’s actual feed intake and its expected feed requirements for maintenance and growth, with low-RFI animals being more efficient at converting forage intake into kilograms of production than high-RFI animals. This study evaluated the impacts of cow age and RFI on body weight (BW) and body condition score (BCS) change, supplement intake, grazing behavior, and resource use of grazing beef cattle grazing mixed-grass rangelands. Heifer post-weaning RFI had little effect on subsequent performance (BW or BCS), grazing behavior, supplement intake behavior, or resource use. However, cow age significantly influenced subsequent performance, grazing behavior, supplement intake behavior, and resource use. In summary, post-weaning RFI had minimal effects on beef cattle performance, grazing behavior, or resource utilization; however, cow age impacted both grazing behavior and resource use.

**Abstract:**

The objectives of this study were to evaluate the influence of RFI and cow age on the supplement intake and grazing behavior of beef cattle. Average daily supplement intake (kg/cow/d) displayed an RFI × cow age interaction (*p* < 0.01), with a linear increase in average daily supplement intake with increasing RFI of 3-year-old cows (*p* < 0.01). Average daily supplement intake (g ∙ kg BW^−1^ ∙ d^−1^) displayed an RFI × cow age interaction (*p* < 0.01), with a quadratic effect on supplement intake of 3-year-old cows (*p* = 0.01). Cow age displayed a quadratic effect on variation of supplement intake (*p* < 0.01), where 1-year-old cows had a greater CV of supplement intake than all other cow ages (*p* < 0.01). Distance traveled displayed a cow age × RFI interaction (*p* = 0.02), where high-RFI 5-year-old cows traveled further per day than low 5-year-old RFI cows. The probability of grazing site selection was influenced by cow age (*p* ≤ 0.03). In summary, heifer post-weaning RFI had minimal effects on beef cattle performance, grazing behavior, or resource utilization; however, cow age impacted both grazing behavior and resource use.

## 1. Introduction

The greatest operating cost for commercial cow-calf producers is providing adequate nutrition for animals is where supplemental feed can account for 65% of the annual expenses [1,2,3]. Selection pressure for feed efficient beef cattle that have lower feed intake while maintaining production could have a significant impact on cow-calf profitability [3]. It has been reported that roughly two-thirds of mature cow energy requirements are utilized for maintenance [4,5,6]. However, substantial animal-to-animal variation, independent of body size and growth, exists in maintenance requirements of cattle [7,8,9]. Consideration of the lower maintenance requirements for low-residual-feed-intake (RFI) cattle becomes much more important as cattle move into times of negative energy balance, such as dormant, late-season grazing [10]. Therefore, improving feed efficiency through genetic selection holds significant opportunity for the beef industry.

Currently, RFI is being used as a selection tool for purchasing bulls and/or retention of heifers. Research evaluating the efficacy of using post-weaning RFI values as selection criteria for beef cattle that fit Western rangeland systems is currently lacking. Most RFI studies utilize energy-dense diets and rations focusing on feedlot performance [11]. Research pertaining to RFI in cattle offered forage-based diets is limited [12], with even fewer data available related to beef cattle forage-based production systems [3,10,13]. As a result, more research is needed to evaluate the utility of RFI estimates on the selection of heifers for extensive forage-based systems [10,14,15].

Western US cow-calf production systems rely heavily on rangeland forages to supply nutrients for both cows and calves [16]. The primary goal in a forage-based livestock production system is to obtain optimal animal performance while effectively utilizing the forage resource base [17]. Seasonal nutrient deficiencies associated with dormant rangeland forages often require protein supplementation to maintain animal performance, production, and provide increased economic returns [18,19]. However, the reported effectiveness of protein supplementation programs on grazing beef cattle performance has been inconsistent, likely due to variation in animal-to-animal protein supplement intake behavior [18,19,20]. Recent research has demonstrated that cow performance is related to supplement intake behavior [21], therefore, RFI, a potential proxy for cow efficiency may also be linked to protein supplement intake behavior. However, information relating cow RFI to protein supplement intake behavior does not currently exist.

Although a central aspect of domestic livestock ecosystems, the spatial component of livestock grazing has remained difficult to interpret [22]. Mechanisms that influence grazing distribution of grazing cattle can be characterized as follows: exogenous, the physical environment (e.g., topography [23,24]), or endogenous (e.g., age and experience [23,25,26,27]). Thus, cattle grazing the same pasture can have different grazing distribution patterns [28]. Recent research has demonstrated that low-RFI cows (more efficient) have larger distribution patterns and outperform high-RFI (less-efficient) cows [10,29]. Therefore, it is possible that grazing behavior may vary with cow RFI (efficiency) and cow age while grazing nutrient deficient forages. However, relationships between exogenous factors with endogenous attributes on grazing behavior are less understood [26]. Therefore, the objectives of this study were to evaluate the influence of cow RFI and age on (1) beef cattle performance; (2) supplement intake behavior; and (3) grazing behavior, resource use, and distribution patterns on winter mixed-grass prairie rangelands. We hypothesized that cow RFI and age interact to influence animal performance, supplement intake, and grazing behavior.

## 2. Materials and Methods

The use of animals in this study was approved by the Agricultural Animal Care and Use Committee of Montana State University AACUC #2018-AA12. All animals used in this study were provided by the Montana Agricultural Experiment Stations, and the study was conducted at the Northern Agricultural Research Center in Havre, Montana.

Two consecutive years of winter grazing studies were conducted on non-lactating, pregnant commercial Angus cows to evaluate the influence of RFI and cow age on beef cattle performance, supplement intake behavior, grazing behavior as well as distribution and resource use patterns. This study was conducted at the Montana State University Northern Agriculture Research Center’s Thackeray Ranch (48°21″ N 109°30′ W), located 21 km south of Havre, MT, USA. Bull Hook Creek, a perennial stream, transects the pasture and is used for livestock watering. Vegetation is dominated by Kentucky bluegrass (*Poa pratensis L*.), bluebunch wheatgrass (*Pseudoroegneria spicata* [Pursh] A. Love), and rough fescue (*Festuca scabrella Torr*.; Reference [20]). Available forage biomass of the study area was estimated by clipping ten randomly located plots to 2 cm in height, immediately prior to grazing, using a 0.25 m^2^ plot frame. Samples were placed in a forced-air oven at 55 °C for 72 h and then weighed and recorded to calculate dry matter production (kg ∙ ha^−1^; Table 1). Vegetation samples were weighed individually and composited by year, ground to pass a 1 mm screen in a Wiley mill, and sent to a Dairy One for nutrient analysis (Dairy One, Ithaca, New York).

### 2.1. Animal Performance and Supplement Intake Behavior

A commercial herd of bred Angus cows (205 in year 1 and 203 in year 2) ranging in age from 1 to 10 years old were classified into one of 6 age groups (1, 2, 3, 4, 5 to 7, and ≥ 8 years old) and grazed on rangeland pastures (~1.5 ha ∙ animal unit month^−1^) from mid-October to early-January each year. Individual cow body weight (BW) and body condition scores (BCS) were obtained following a 16-h shrink pre- and post-grazing (Table 2). Body condition scores were based on a 1–9 scale (BCS: 1 = emaciated, 9 = obese, [30]) and assessed by two trained individuals, then averaged for a final BCS. All heifers, (9–11 months of age), went through a 70-day post-weaning RFI trial [7,29] using a GrowSafe system (GrowSafe DAQ 4000E; GrowSafe System Ltd., Airdrie, AB, Canada) and were classified as either low (<−0.50 SD from mean), or high (>+0.50 SD from the mean) RFI within their contemporary age group [31].

A free-choice, self-fed, canola based pressed block supplement (28.7% crude protein (CP; year 1) and 30% CP (year 2)) was provided ad-libitum to cattle. The target daily-recommended intake range was 0.45 to 0.91 kg ∙ cow^−1^ ∙ d^−1^ with 23% salt, texture and bitterness to limit daily intake. Individual animals were equipped with an electronic identification tag attached to the left ear for the measurement of daily individual supplement intake (kg ∙ cow^−1^ ∙ d^−1^; g ∙ kg BW^−1^ ∙ d^−1^), and time spent at supplement feeders (min∙ d^−1^) using centrally located SmartFeed Pro self-feeder systems (C-Lock Inc., Rapid City, South Dakota) with a total of 2 trailers and 8 feeding stations (4 per trailer). Variation in supplement intake, measured as the coefficient of variation (% CV), was based on daily intake estimates for individual animals.

### 2.2. Grazing Behavior and Resource Use

Each year, a subset of 30 cows were randomly selected within age (2, 5, and 8 years old) and RFI (Low, High) and fitted with Lotek GPS collars containing an activity sensor (Lotek Engineering, Newmarket, ON, Canada; 5 collars per RFI × cow age combinations) representing a minimum of 39% of the total cattle population within each RFI × age combination each year. GPS collars were configured to record positions at 15-min intervals, and activity sensor measurements at 5-min intervals to determine distance traveled, location, and duration of grazing activities, as well as resource use [32,33,34]. Due to limited battery life of GPS collars, supplement intake, grazing behavior and resource use was only measured during the last 45 days of grazing each year. Individual cow was considered the experimental unit to evaluate effects of age and RFI on supplement intake behavior. Grazing activities were derived by the binary classification methods developed by Augustine and Derner [35] to evaluate time spent grazing and foraging distribution. Observations were limited to grazing to determine critical foraging areas rather than general occupancy [17,26]. Pasture supplement and water locations were recorded by using a handheld GPS unit (spatial error < 10-m). Spatial layers including aspect, terrain ruggedness (sum change in elevation between a grid cell and its eight neighboring cells; Reference [36]) and distance from supplement and water locations were developed by using the spatial analysis tool in ArcGIS (Environmental Systems Research Institute, Redlands, CA, USA) and a digital elevation model at a 30 m^2^ resolution.

### 2.3. Statistical Analysis

The influence of RFI and cow age on cow BCS, BW (Supplementary Materials Table S1), supplement intake behavior (Supplementary Materials Table S2), time spent grazing, and distance traveled (Supplementary Materials Table S3) were analyzed by using a generalized linear mixed model, with a Gaussian error structure, in an ANOVA framework that included RFI, age, and the interactions of RFI and age as fixed effects, and individual cow and year as random effects. An alpha ≤ 0.05 was considered significant with animal considered as the experimental unit. Linear and quadratic effects were determined, using orthogonal polynomial contrasts for each analysis. The Tukey method was used to separate means when *p* < 0.05. Tendencies were considered when *p* ≤ 0.10.

To model relative resource selection for cattle grazing late season dormant forages, individual GPS-collared cows were defined as the biological unit of interest. To evaluate the response of an individual cow’s space use to pasture level covariates, we used multiple regression in a resource utilization function (RUF) analysis with the ruf.fit package in R [37,38,39]. Resource utilization functions reduce error associated with location estimation and increase sensitivity for detecting resource selection by evaluating within animal variation in resource use and incorporating an individual cow’s entire grazing distribution, independently, while accounting for spatial autocorrelation [37,38,40].

Due to cattle grazing distribution being defined by pasture boundary, GPS grazing locations were used to build RUFs to quantify cow selection of environmental covariates within pasture (third-order scale; [41]). Specific utilization density rasters for grazing locations were created for each individual at a 30 m^2^ resolution, using the adehabitatHR and raster packages in R [42,43]. Relative use values were bound between 1 and 99 for each 30 m^2^ cell based off of the relative volume of use within the cell compared to all other cells in the pasture [37]. Pasture level spatial covariates anticipated to effect resource utilization included distance to supplement and water, elevation, terrain ruggedness and aspect. Individual relative use and pasture level covariates rasters were stacked and converted to data files, using the raster function in R as input for the ruf.fit package (see Supplementary materials Table S4) [39,40]. To meet the assumptions of multiple regression models, individual relative use values were log-transformed. Standardized β coefficients were developed and evaluated for each cow to determine the influence of the pasture level covariates on cattle resource utilization [37,39,40].

Significant predictors of resource use were determined by standardized coefficients with 95% confidence intervals not overlapping zero [37,38]. Significant resource utilization coefficients were determined to be greater or less than expected based on availability of the covariate within the pasture [35,36]. For pasture level covariates displaying high herd-level variability in grazing resource utilization (herd-level SE of standardized coefficients > 0.25; [44]), a post hoc analysis was conducted to evaluate the effects of RFI, cow age, and the interaction of RFI and cow age on resource use coefficients relative to each pasture covariate, using ANOVA with a generalized linear mixed model, with a Gaussian error structure, including year as a random intercept. An alpha ≤ 0.05 was considered significant with animal considered as the experimental unit. For age main effects, linear and quadratic effects were determined by using orthogonal polynomial contrasts for each analysis. The Tukey method was used to separate means when *p* < 0.05. Tendencies were considered when *p* ≤ 0.10.

**Figure 1:**
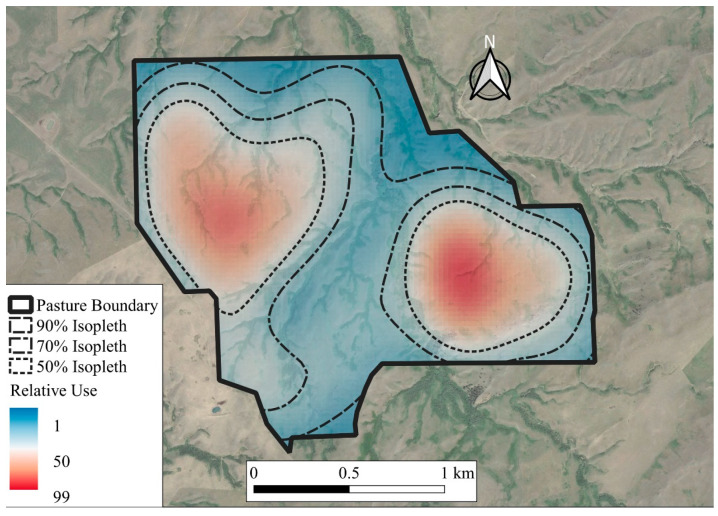
Isopleth showing relative use of one cow winter grazing dormant mixed-grass prairie at the Northern Agriculture Research Center’s Thackeray Ranch, Havre, MT, USA (45-day grazing period).

Core grazing areas for individual cows were estimated by using the kernel utilization distribution function in the adehabitatHR package in R [42] (see Figure 1). Kernel utilization distributions are three-dimensional representations of estimated distribution of use, used to calculate home range [45]. The estimated values of use for the kernel utilization distributions containing only grazing locations were then used to create a contour representing 50% of the volume of use delineating core grazing areas [46,47,48]. The area of the 50% contours were calculated by using the gArea function in the rgeos package of R (see Supplementary Materials Table S5) [49]. Due to pasture management unit defining the extent of core grazing areas of cattle, contours representing core grazing areas were bound by pasture boundary. The influence of RFI and cow age on core grazing area was analyzed by using a generalized linear mixed model, with a Gaussian error structure, in an ANOVA framework including RFI, age, and the interactions of RFI and age as fixed effects, and year as a random intercept. An alpha ≤ 0.05 was considered significant with animal considered as the experimental unit. For age main effects, linear and quadratic effects were determined by using orthogonal polynomial contrasts for each analysis. The Tukey method was used to separate means when *p* < 0.05. Tendencies were considered when *p* ≤ 0.10. All statistical analyses were performed in R [50].

## 3. Results

### 3.1. Supplement Intake Behavior and Animal Performance

Body weight change over the 84-day grazing period exhibited a tendency for an RFI × cow age interaction (*p* = 0.08). Specifically, 4-year-old cows BW change decreased linearly with increasing RFI (*p* < 0.01), averaging 22.69 ± 23.66, 2.59 ± 23.31, and −12.49 ± 23.60 kg weight change for low, average, and high RFI, respectively. No RFI differences (*p* > 0.44) were observed for change of cow BW within all other cow ages. Cow age influenced (*p* = 0.02) weight change, over the 84-day grazing period; however, no linear or quadratic responses were noted (*p* ≥ 0.19) with weight changes ranging from −5.01 ± 22.87 kg (yearlings) to 8.72 ± 22.92 kg (3-year-old cows) with all other cow age being intermediate. Change in BCS exhibited a tendency (*p* = 0.07) for a quadratic effect of cow age (*p* = 0.01), with 1-year-old cows losing BCS (−0.10 ± 0.10 units) compared to 4-year-old cows gaining BCS (0.15 ± 0.10 units) with all other cow age being intermediate over the 84-day grazing period.

**Figure 2:**
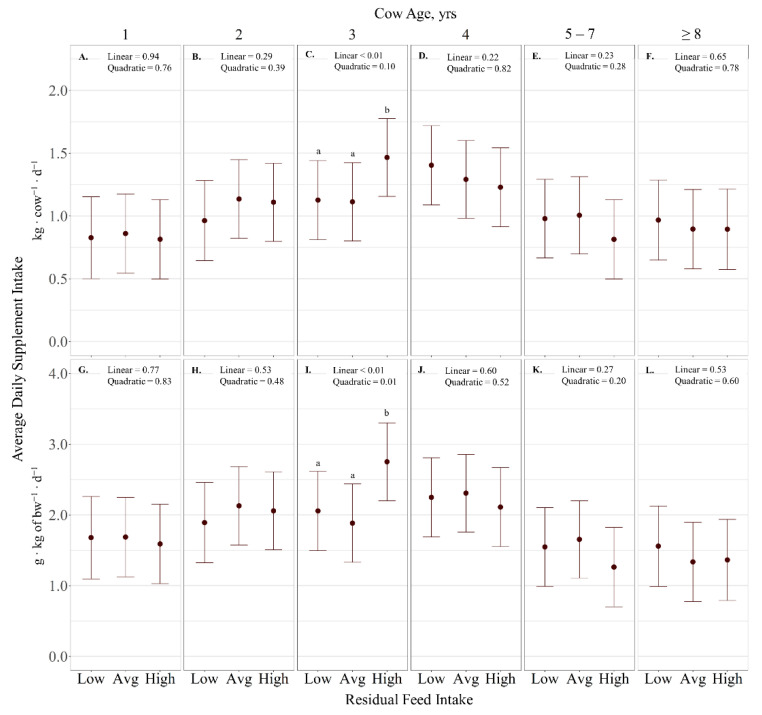
Influence of RFI × cow age (*p* < 0.01) on average daily supplement intake (expressed as g ∙ kg^−1^ of BW ∙ d^−1^ (**A**–**F**), as well as kg ∙ cow^−1^ ∙ d^−1^ (**G**–**L**) ± SE) by cattle grazing dormant mixed-grass prairie in 2018/2019 and 2019/2020 at the Northern Agriculture Research Center’s Thackeray Ranch, Havre, MT, USA. Means lacking common letters (a, b) differ (*p* < 0.05).

**Figure 3:**
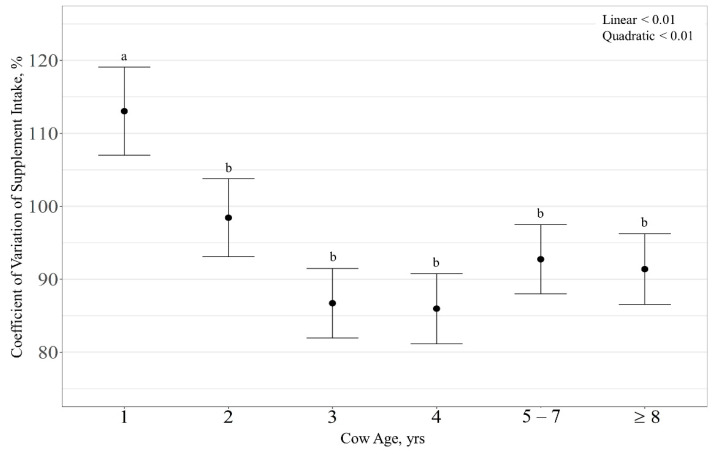
Influence of cow age on coefficient of variation of supplement intake (expressed as % ± SE) by cattle grazing dormant northern mixed-grass prairie in 2018/2019 and 2019/2020 at the Northern Agriculture Research Center’s Thackeray Ranch, Havre, MT, USA. Means lacking common letters (a, b) differ (*p* < 0.05).

Average daily supplement intake (kg ∙ cow^−1^ ∙ d^−1^) displayed an RFI × cow age interaction (*p* < 0.01; Figure 2), with RFI exhibiting a linear effect on 3-year-old cows (*p* < 0.01) where supplement intake increased with increasing RFI (Figure 2C). However, RFI had no effect on supplement intake for other cow age (*p* > 0.28). Supplement intake expressed as g ∙ kg^−1^ BW^−1^ ∙ d^−1^ displayed an RFI × cow age interaction (*p* < 0.01). Specifically, a quadratic effect of RFI was observed on supplement intake of 3-year-old cows (*p* = 0.01; Figure 2I), where high-RFI cattle consumed more supplement than low- and average-RFI cattle (*p* < 0.01). However, RFI had no effect on supplement intake for other cow ages (*p* > 0.23). Likewise, RFI effects were not observed for variation of supplement intake (% CV; *p* > 0.69). However, there was a quadratic effect of cow age on variation in supplement intake (*p* < 0.01; Figure 3), where 1-year-old cows had a larger CV of supplement intake than all other ages (*p* < 0.01).

**Figure 4:**
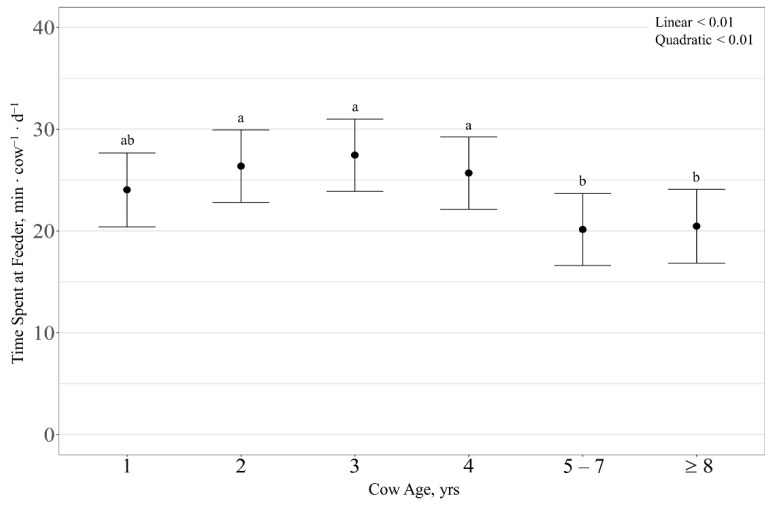
Influence of cow age on average daily time spent at supplement feeder (expressed as minutes ∙ cow^−1^ ∙ d^−1^ ± SE) by cattle grazing dormant northern mixed-grass prairie in 2018/2019 and 2019/2020 at the Northern Agriculture Research Center’s Thackeray Ranch, Havre, MT, USA. Means lacking common letters (a, b) differ (*p* < 0.05).

There was no effect (*p* > 0.13) of RFI or cow age observed on supplement intake rate, with supplement intake rate averaging 60.6 ± 32.0 g ∙ min^−1^. Time spent at the feeders was quadratically influenced (*p* < 0.01) by cow age, where 2-, 3-, and 4-year-old cows spent more time at the feeders than 1-, 5–7-, and ≥8-year-old cows (*p* < 0.02; Figure 4). However, time spent at the feeders exhibited a tendency for an RFI × cow age interaction (*p* = 0.08), with a quadratic effect (*p* = 0.03) of RFI for 5–7-year-old cows, where average-RFI cows tended to spend more time at the feeders than high-RFI cows (*p* = 0.08).

### 3.2. Grazing Behavior and Resource Use

**Figure 5:**
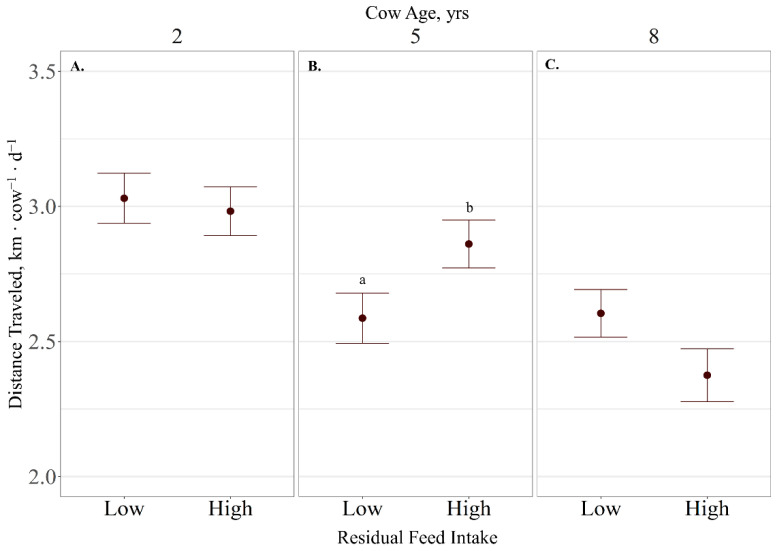
Influence of residual feed intake × cow age (**A**–**C**; *p* = 0.02) on average distance traveled expressed as km cow^−1^ ∙ d^−1^ ± SE) by cattle grazing dormant northern mixed-grass prairie in 2018/2019 and 2019/2020 at the Northern Agriculture Research Center’s Thackeray Ranch, Havre, MT, USA. Means lacking common letters (a, b) differ (*p* < 0.05).

Distance traveled displayed a cow age × RFI interaction (*p* = 0.02), where high-RFI 5-year-old cows traveled further per day than low-RFI 5-year-old cows (*p* < 0.03), and low-RFI 8-year-old cows tended (*p* = 0.08; Figure 5) to travel further than high-RFI 8-year-old cows. No RFI effects (*p* < 0.71) were observed on distance traveled for 2-year-old cows. Distance traveled ranged from 3.00 ± 0.09 for 2-year-old cows to 2.49 ± 0.09 km ∙ d^−1^ for 8-year-old cows. Time spent grazing was not influenced by cow age (*p* = 0.29) nor RFI (*p* = 0.40), averaging 6.06 ± 0.44 h ∙ d^−1^.

**Figure 6:**
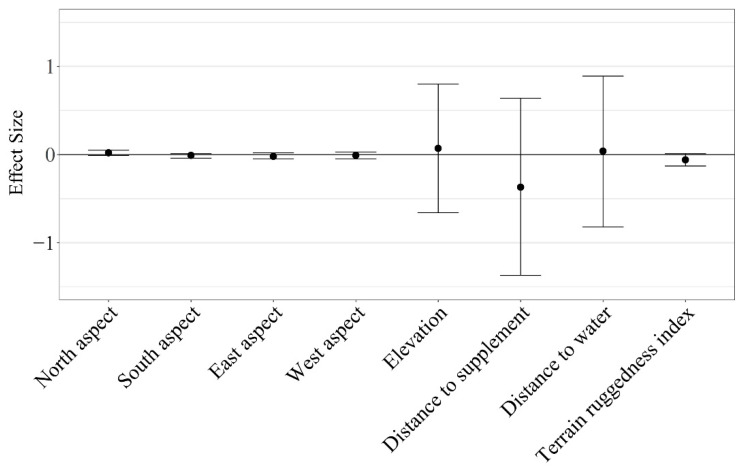
Mean standardized herd-level effect size ($\hat{\bar{\beta }}$ ± 95% CI) for cattle grazing dormant northern mixed-grass prairie in 2018/2019 and 2019/2020 at the Northern Agriculture Research Center’s Thackeray Ranch, Havre, MT, USA.

**Figure 7:**
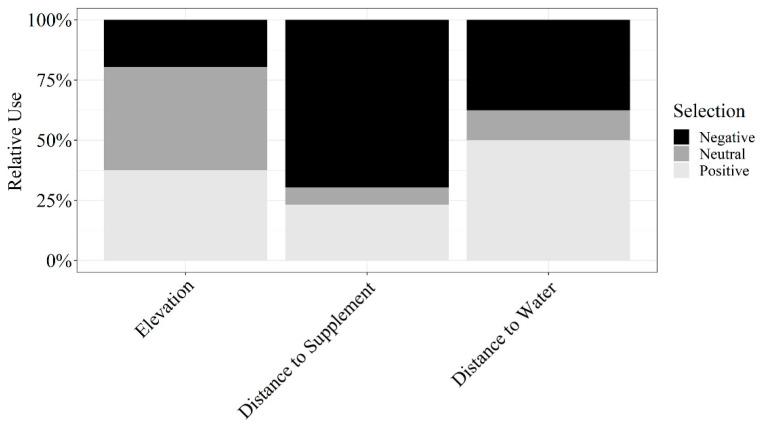
Influence of elevation, distance to supplement, and distance to water on relative use by cattle grazing dormant northern mixed-grass prairie in 2018/2019 and 2019/2020 at the Northern Agriculture Research Center’s Thackeray Ranch, Havre, MT, USA.

Herd-level grazing resource utilization for cattle on dormant rangeland forage was not impacted by aspect ($\hat{\bar{\beta }}$ North = 0.02 ± 0.01; $\hat{\bar{\beta }}$ South = −0.01 ± 0.01; $\hat{\bar{\beta }}$ East = −0.02 ± 0.02; $\hat{\bar{\beta }}$ West = −0.01 ± 0.02), elevation ($\hat{\bar{\beta }}$ = 0.07 ± 0.37), distance to supplement ($\hat{\bar{\beta }}$ = −0.37 ± 0.51), distance to water ($\hat{\bar{\beta }}$ = 0.04 ± 0.44), or terrain ruggedness ($\hat{\bar{\beta }}$ = −0.06 ± 0.03; Figure 6). However, resource utilization relative to elevation, distance to supplement, and distance to water were highly variable among individuals (herd-level SE of standardized coefficients > 0.25; Figure 7). Therefore, we conducted a post hoc analysis evaluating the effects of RFI, cow age, and the interaction of RFI and cow age on grazing resource utilization relative to elevation, distance to supplement, and distance to water.

**Figure 8:**
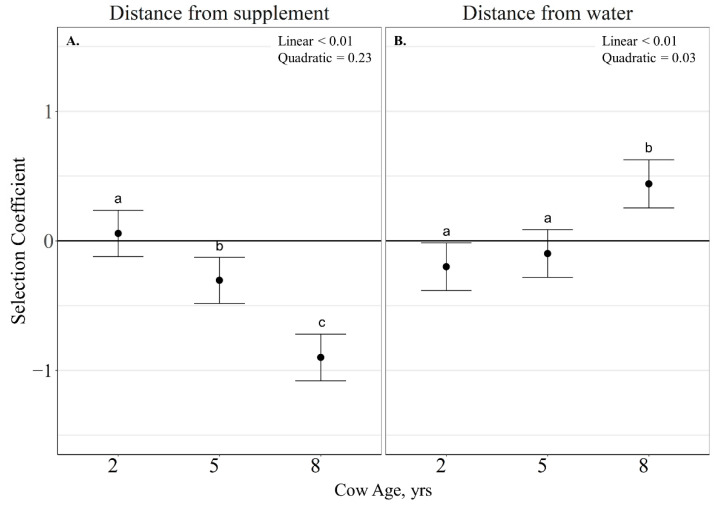
Influence of cow age on distance from supplement (**A**) and distance from water (**B**; expressed as slope estimate ± CI) of cattle grazing dormant northern mixed-grass prairie in 2018/2019 and 2019/2020 at the Northern Agriculture Research Center’s Thackeray Ranch, Havre, MT, USA. Means lacking common letters (a, b) differ (*p* < 0.05).

Cow age did not interact with RFI (*p* > 0.11) when evaluating the probability of grazing site selection relative to elevation, distance from supplement and water. As a result, only RFI and cow age main effects are reported. The probability of grazing site selection relative to elevation was not influenced by RFI (*p* = 0.62) or cow age (*p* = 0.16). The probability of grazing site selection relative to distance to supplement was not impacted by RFI (*p* = 0.68), but was linearly influenced by cow age ($\hat{\bar{\beta }}$ 2-years old = 0.06 ± 0.08; $\hat{\bar{\beta }}$ 5-years old = −0.31 ± 0.08; $\hat{\bar{\beta }}$ 8-years old = −0.90 ± 0.08; *p* < 0.01) with older cows grazing closer to supplement locations than younger cows (Figure 8A). The probability of grazing site selection relative to distance to water was also not impacted by RFI (*p* = 0.63); however, it was quadratically influenced by cow age (($\hat{\bar{\beta \;}}$2-years old = −0.20 ± 0.08; ($\hat{\bar{\beta \;}}$5-years old = −0.10 ± 0.08; ($\hat{\bar{\beta }}\;$8-years old = 0.44 ± 0.08; *p* = 0.03) with 8-year-old cows selecting grazing locations further from water than 2- and 5-year-old cows (Figure 8B).

**Figure 9:**
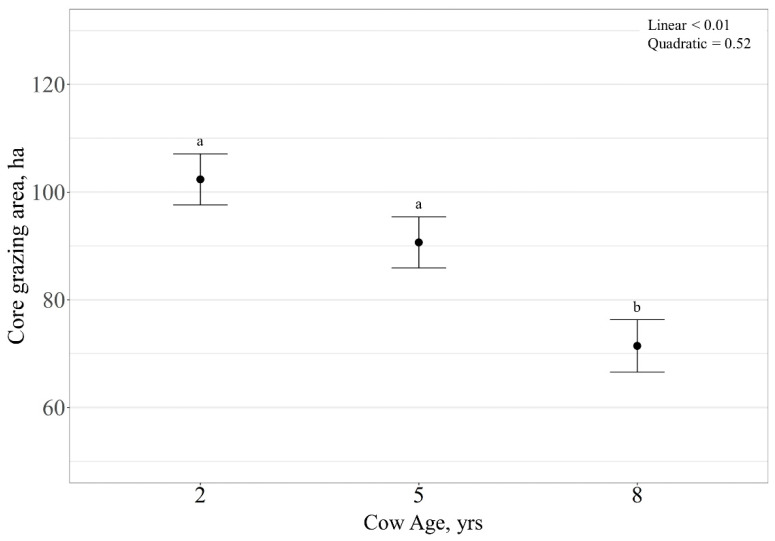
Influence of cow age on core grazing area (expressed as ha ± SE) by cattle grazing dormant northern mixed-grass prairie in 2018/2019 and 2019/2020 at the Northern Agriculture Research Center’s Thackeray Ranch, Havre, MT, USA. Means lacking common letters (a, b) differ (*p* < 0.05).

Core grazing area (ha) was linearly affected by cow age (*p* < 0.01), with core grazing area decreasing with increasing cow age (Figure 9). There was no effect of RFI on core grazing area (*p* = 0.71), but a tendency for a cow age × RFI interaction was observed (*p* = 0.07). However, there were no differences observed for RFI within cow age (*p* > 0.10) relative to size of core grazing area.

## 4. Discussion

While the estimation of RFI on young bulls and developing heifers is becoming a more popular practice in the US beef industry, the use of RFI of post-weaned heifers as a selection criterion for retention of replacement females for forage-based rangeland environments has not been well studied. Conventional wisdom suggests that cattle with low RFI values are more efficient in converting nutrients to maintenance energy and body weight gain. Few research experiments have investigated the effects of RFI on beef cattle performance, supplement intake and grazing behavior or resource use while grazing late season dormant rangelands. If low-RFI cattle have lower maintenance requirements, selection for low-RFI cattle on low quality late-season mature rangelands could lead to cattle that are more efficient while grazing on low-energy and low-protein diets, assuming that low-RFI cattle have lower maintenance requirements when facing nutritionally stressful periods.

Sprinkle and coworkers [10] were the first to examine the effects of RFI on livestock performance while grazing low-quality dormant forages; however, their research focused solely on 2-year-old cows. In contrast, our study focused on the effects of RFI across multiple age groups on supplement intake behavior, grazing behavior, and resource use while grazing low quality dormant mixed-grass rangelands. Most of the research investigating the effects of RFI on cattle performance has occurred on both irrigated and improved dryland summer pastures [11,14,51]. All the above-mentioned research trials can be characterized as having adequate forage quality to meet maintenance requirements with the exception of Sprinkle et al. [10], where their grazing and forage conditions were similar to those experienced during our research trial.

Previous research [29] reported that low-RFI cows grazing summer rangelands in central Idaho travel further and graze longer than high-RFI cows at warmer temperatures. Conversely, we observed no effect of RFI on grazing behavior, and resource use during our late fall/early winter grazing trial. However, 5-year-old high-RFI cows did travel further than low-RFI 5-year-old cows, and low-RFI 8-year-old cows tended to travel further than high-RFI 8-year-old cows with no observed differences among 2-year-old cows. Sprinkle et al. [10] reported that, while grazing late season dormant rangelands in Idaho, low-RFI 2-year-old cows lost less weight and body condition compared to high-RFI 2-year-old cows with no difference in daily distance traveled or foraging rate (bites ∙ min^−1^). Conversely, we observed a decrease in BW change with increasing RFI for 4-year-old cows, with no observed changes in cow BW within all other cow ages. However, we did not observe effects of RFI on BCS change, and the differences we observed in distance traveled and core grazing area were associated with cow age and not RFI. Similarly, Sprinkle et al. [52] also reported no difference in distance traveled or time spent grazing between low-RFI and high-RFI 2-year-old cows grazing supplemented fall pastures in central Idaho. Our results are also consistent with Meyer et al. [3], where RFI did not affect BW and BCS change or supplement intake while grazing during late winter and early spring.

Although limited, previous experiments evaluating supplement intake of mixed-age beef herds have reported that younger cows spent less time at the supplement feeders and consumed less supplement than older cows [53,54]. In contrast, Wyffels et al. [44] reported that younger cattle consumed more supplement and visited the supplement feeders more often than older cows. Results from our research are similar to Wyffels et al. [44], since we observed a quadratic effect in supplement intake related to cow age, where 1-year-old cows consumed more and had a larger CV of supplement intake than older cows.

Previous research reported that cow age significantly impacted grazing behavior and distribution with older cows grazing further from water and using higher elevations than younger cows [26,55]. Our results agree with these reports, where older cows selected grazing locations further from water than younger cows. Wyffels et al. [55] reported results similar to our study, where older cows selected grazing locations closer to supplement feeders and herd-level resource utilization was negatively related to terrain ruggedness on mixed-grass prairie rangelands. In our study we observed that herd-level resource utilization of cattle grazing dormant mixed-grass prairie rangelands was not impacted by aspect, elevation, or terrain ruggedness.

Our research suggests that in dormant forage grazing environments, heifer post-weaning RFI has little effect on supplement intake behavior (with the exception of 3-year-old cows), grazing behavior, or resource use. We observed no RFI impact on BW or BCS change over the 84-day grazing period, with the exception of 4-year-old cows. Our results agree with previous research [56] that suggests post-weaning RFI is independent of mature cow BW and may have little impact on the efficiency of cattle winter grazing on dormant rangelands. However, despite the lack of differences in supplement intake behavior, grazing behavior, and resource use in our study, if cows selected for low RFI have the same performance parameters as high-RFI cows while consuming less feed, selection for low-RFI cattle would still be warranted. As a result, further research is needed to investigate the relationship of heifer post-weaning RFI and dry-matter intake of cows at different ages and stages of production.

## 5. Conclusions

Heifer post-weaning RFI had little effect on subsequent cow performance (BW or BCS), grazing behavior, supplement intake behavior, and resource use. However, cow age significantly influenced cow performance, grazing behavior, supplement intake behavior, and resource use. We also observed high individual variability in grazing site selection, suggesting that individual-level factors may be driving grazing resource use and grazing behavior. Therefore, our research suggests that cow age has more of an impact on resource use, supplement intake, and grazing behavior than heifer post-weaning RFI while grazing dormant-season mixed-grass prairie rangelands.

## Figures and Tables

**Table 1:** Average yearly grass available biomass (kg DM ∙ ha^−1^), crude protein (CP% DM), acid detergent fiber (ADF %), neutral detergent fiber (NDF %), and total digestible nutrients (TDN %) of the experimental pasture for the 2 years of grazing (2018/2019 and 2019/2020) at the Northern Agricultural Research Center Thackeray Ranch, Havre, MT, USA.

	Grass Production (kg/ha)	CP (%)	ADF (%)	NDF (%)	TDN (%)
Year 1	1790	5.4	41.9	63.2	56.0
Year 2	1456	5.4	39.9	66.9	55.0

**Table 2:** Initial body weight (BW) and body condition score (BCS) for 6 age classes of cattle (±SE) across a 2-year grazing trial (2018/2019 and 2019/2020) at the Northern Agricultural Research Center Thackeray Ranch, Havre, MT, USA.

	Age Class					
	1	2	3	4	5–7	≥8
Cow number						
Year 1	37	34	28	30	46	30
Year 2	33	28	30	24	47	41
Initial BW, kg						
Year 1	489.6 ± 5.30	495.3 ± 7.55	565.4 ± 11.04	597.0 ± 8.88	617.2 ± 8.72	610.6 ± 9.28
Year 2	467.5 ± 4.20	508.4 ± 7.05	561.0 ± 9.16	616.7 ± 12.41	637.5 ± 6.75	624.6 ± 8.27
Initial BCS ^1^						
Year 1	5.76 ± 0.04	5.24 ± 0.08	5.52 ± 0.11	5.59 ± 0.09	5.56 ± 0.08	5.59 ± 0.07
Year 2	5.76 ± 0.05	5.21 ± 0.05	5.16 ± 0.11	5.47 ± 0.09	5.52 ± 0.06	5.41 ± 0.07
Final BW, kg						
Year 1	497.0 ± 5.84	524.4 ± 7.11	496.4 ± 10.05	624.9 ± 6.36	644.3 ± 7.70	641.8 ± 9.39
Year 2	448.7 ± 4.28	490.6 ± 7.04	547.7 ± 7.58	595.8 ± 11.64	606.8 ± 7.02	609.9 ± 8.24
Final BCS						
Year 1	5.66 ± 0.04	5.06 ± 0.10	5.41 ± 0.09	5.63 ± 0.05	5.61 ± 0.06	5.45 ± 0.07
Year 2	5.67 ± 0.05	5.29 ± 0.05	5.39 ± 0.07	5.69 ± 0.09	5.65 ± 0.06	5.57 ± 0.07

^1^ Body condition score based on a 1–9 scale: 1 = emaciated, 9 = obese.

## Data Availability

The data presented in this study are available in Supplementary Material Tables S1–S5.

## Supplementary Materials

The following are available online at https://www.mdpi.com/article/10.3390/ani11061518/s1. Table S1: Cow body weight and body condition score change data. Table S2: Supplement intake behavior data. Table S3: Time spent grazing and distance traveled data. Table S4: Resource utilization data. Table S5: Grazing core area data.

## References

[1] Arthur P.F., Archer J.A., Herd R.M. (2004). Feed intake and efficiency in beef cattle: Overview of recent Australian research and challenges for the future. Aust. J. Exp. Agric..

[2] Van der Westhuizen R.R., Van der Westhuizen J., Schoeman S.J. (2004). Genetic variance components of residual feed intake and feed conversion ratio and their correlations with other production traits in beef bulls. S. Afr. J. Anim. Sci..

[3] Meyer A.M., Kerley M.S., Kallenbach R.L. (2008). The effect of residual feed intake classification on forage intake by grazing beef cows. J. Anim. Sci..

[4] Ferrell C.L., Jenkins T.G. (1985). Energy utilization by Hereford and Simmental males and females. Anim. Sci..

[5] Ferrell C.L., Jenkins R.G. (1988). Influence of biological types on energy requirements. Beef Res. Prog. Rep..

[6] Montaño-Bermudez M., Nielsen M.K., Deutscher G.H. (1990). Energy requirements for maintenance of crossbred beef cattle with different genetic potential for milk. J. Anim. Sci..

[7] Arthur P.F., Archer J.A., Johnston D.J., Herd R.M., Richardson E.C., Parnell P.F. (2001). Genetic and phenotypic variance and covariance components for feed intake, feed efficiency, and other postweaning traits in Angus cattle. J. Anim. Sci..

[8] Nkrumah J.D., Okine E.K., Mathison G.W., Schmid K., Li C., Basarab J.A., Price M.A., Wang Z., Moore S.S. (2006). Relationships of feedlot feed efficiency, performance, and feeding behavior with metabolic rate, methane production, and energy partitioning in beef cattle. J. Anim. Sci..

[9] Crowley J.J., McGee M., Kenny D.A., Crews D.H., Evans R.D., Berry D. (2010). Phenotypic and genetic parameters for different measures of feed efficiency in different breeds of Irish performance-tested beef bulls. J. Anim. Sci..

[10] Sprinkle J.E., Taylor J.B., Clark P.E., Hall J.B., Strong N.K., Roberts-Lew M.C. (2020). Grazing behavior and production characteristics among cows differing in residual feed intake while grazing late season Idaho rangelands. J. Anim. Sci..

[11] Lawrence P., Kenny D.A., Earley B., Crews D.H., McGee M. (2011). Grass silage intake, rumen and blood variables, ultrasonic and body measurements, feeding behavior, and activity in pregnant beef heifers differing in phenotypic residual feed intake. J. Anim. Sci..

[12] Arthur P.F., Herd R.M., Wilkins J.F., Archer J.A. (2005). Maternal productivity of Angus cows divergently selected for post-weaning residual feed intake. Aust. J. Exp. Agric..

[13] Basarab J.A., McCartney D., Okine E.K., Baron V.S. (2007). Relationships between progeny residual feed intake and dam productivity traits. Can. J. Anim. Sci..

[14] Manafiazar G., Basarab J.A., Baron V.S., McKeown L., Doce R.R., Swift M., Undi M., Wittenberg K., Ominski K. (2015). Effect of post-weaning residual feed intake classification on grazed grass intake and performance in pregnant beef heifers. Can. J. Anim. Sci..

[15] Kenny D.A., Fitzsimons C., Waters S.M., McGee M. (2018). Invited review: Improving feed efficiency of beef cattle—The current state of the art and future challenges. Animal.

[16] Galyean M.L., Goetsch A.L., Jung H.G., Buxton D.R., Hatfield R.D., Ralph J. (2015). Utilization of Forage Fiber by Ruminants. Forage Cell Wall Structure and Digestibility.

[17] Wyffels S.A., Petersen M.K., Boss D.L., Sowell B.F., Bowman J.G., McNew L.B. (2019). Dormant Season Grazing: Effect of Supplementation Strategies on Heifer Resource Utilization and Vegetation Use. Rangel. Ecol. Manag..

[18] Bowman J.G.P., Sowell B.F. (1997). Delivery method and supplement consumption by grazing ruminants: A review. J. Anim. Sci..

[19] DelCurto T., Hess B.W., Huston J.E., Olson K.C. (2000). Optimum supplementation strategies for beef cattle consuming low-quality roughages in the western United States. J. Anim. Sci..

[20] Wyffels S.A., Parsons C.T., Dafoe J.M., Boss D.L., McClain T.P., Carter B.H., DelCurto T. (2020). The influence of age and winter environment on Rumax Bovibox and Bovibox HM supplement intake behavior of winter grazing beef cattle on mixed-grass rangelands. Transl. Anim. Sci..

[21] Wesley R.L., Cibils A.F., Mulliniks J.T., Pollak E.R., Petersen M.K., Fredrickson E.L. (2012). An assessment of behavioural syndromes in rangeland-raised beef cattle. Appl. Anim. Behav. Sci..

[22] Coughenour M.B. (1991). Invited Synthesis Paper: Spatial Components of Plant-Herbivore Interactions in Pastoral, Ranching, and Native Ungulate Ecosystems. J. Range Manag..

[23] Bailey D.W., Gross J.E., Laca E.A., Rittenhouse L.R., Coughenour M.B., Swift D.M., Sims P.L. (1996). Mechanisms That Result in Large Herbivore Grazing Distribution Patterns. J. Range Manag..

[24] Beaver J., Olson B. (1997). Winter range use by cattle of different ages in southwestern Montana. Appl. Anim. Behav. Sci..

[25] Dunn R.W., Havstad K.M., Ayers E.L. (1988). Grazing behavior response of rangeland beef cattle to winter ambient temperature and age. App. Anim. Behav. Sci..

[26] Walburger K.J., Wells M., Vavra M., DelCurto T., Johnson B.K., Coe P. (2009). Influence of Cow Age on Grazing Distribution in a Mixed-Conifer Forest. Rangel. Ecol. Manag..

[27] Launchbaugh K.L., Howery L.D. (2005). Understanding Landscape Use Patterns of Livestock as a Consequence of Foraging Behavior. Rangel. Ecol. Manag..

[28] Bailey D.W. (2004). Management strategies for optimal grazing distribution and use of arid rangelands. J. Anim. Sci..

[29] Sprinkle J.E., Ellison M.J., Hall J.B., Yelich J.V., Willmore C.M., Brennan J.R. (2019). Are low-residual feed intake cows adapted to rangelands?. Transl. Anim. Sci..

[30] Wagner J.J., Lusby K.S., Oltjen J.W., Rakestraw J., Wettemann R.P., Walters L.E. (1988). Carcass Composition in Mature Hereford Cows: Estimation and Effect on Daily Metabolizable Energy Requirement During Winter. J. Anim. Sci..

[31] Parsons C.T., Dafoe J.M., Wyffels S.A., Van Emon M., DelCurto T., Boss D.L. (2021). Impacts of heifer post-weaning residual feed intake classification on reproductive and performance measurements of first, second and third parity Angus beef females. Transl. Anim. Sci..

[32] Turner L., Udal M., Larson B.T., Shearer S. (2000). Monitoring cattle behavior and pasture use with GPS and GIS. Can. J. Anim. Sci..

[33] Ungar E.D., Henkin Z., Gutman M., Dolev A., Genizi A., Ganskopp D. (2005). Inference of Animal Activity from GPS Collar Data on Free-Ranging Cattle. Rangel. Ecol. Manag..

[34] Brosh A., Henkin Z., Ungar E.D., Dolev A., Shabtay A., Orlov A., Yehuda Y., Aharoni Y. (2010). Energy cost of activities and locomotion of grazing cows: A repeated study in larger plots1. J. Anim. Sci..

[35] Augustine D.J., Derner J.D. (2013). Assessing Herbivore Foraging Behavior with GPS Collars in a Semiarid Grassland. Sensors.

[36] Riley S.J. (1999). Index that quatifies topographic heterogeneity. Intrmntn. J. Sci..

[37] Marzluff J.M., Millspaugh J.J., Hurvitz P., Handcock M.S. (2004). Relating resources to a probabilistic measure of space use: Forest fragments and Steller’s jays. Ecology.

[38] Winder V.L., McNew L.B., Gregory A.J., Hunt L.M., Wisely S.M., Sandercock B.K. (2014). Space use by female Greater Prairie-Chickens in response to wind energy development. Ecosphere.

[39] Handcock M.S. (2015). Estimates of the Resource Utilization Function. Version 1.5-3. http://www.csde.washington.edu/~handcock/ruf.

[40] Kertson B.N., Marzluff J.M. (2010). Improving studies of resource selection by understanding resource use. Environ. Conserv..

[41] Johnson D.H. (1980). The Comparison of Usage and Availability Measurements for Evaluating Resource Preference. Ecology.

[42] Calenge C. (2006). The package “adehabitat” for the R software: A tool for the analysis of space and habitat use by animals. Ecol. Model..

[43] Hijmans R.J. (2019). Raster: Geographic Data Analysis and Modeling. R Package Version 2.8-19. https://CRAN.R-project.org/package=raster.

[44] Wyffels S.A., Boss D.L., Sowell B.F., DelCurto T., Bowman J.G.P., McNew L.B. (2020). Dormant season grazing on northern mixed grass prairie agroecosystems: Does protein supplement intake, cow age, weight and body condition impact beef cattle resource use and residual vegetation cover?. PLoS ONE.

[45] Clapp J.G., Beck J.L. (2015). Evaluating distributional shifts in home range estimates. Ecol. Evol..

[46] Heupel M.R., Simpfendorfer C.A., Hueter R.E. (2004). Estimation of Shark Home Ranges using Passive Monitoring Techniques. Environ. Biol. Fishes.

[47] Kie J.G., Matthiopoulos J., Fieberg J., Powell R.A., Cagnacci F., Mitchell M.S., Gaillard J.-M., Moorcroft P.R. (2010). The home-range concept: Are traditional estimators still relevant with modern telemetry technology?. Philos. Trans. R. Soc. B Biol. Sci..

[48] Garitano-Zavala A., Chura Z., Cotín J., Ferrer X., Nadal J. (2013). Home range extension and overla-1of the Ornate Tinamou (*Nothoprocta ornata)* in an Andean agro-ecosystem. Wilson J. Ornith..

[49] Bivand R., Rundel C. (2020). Rgeos: Interface to Geometry Engine—Open Source (‘GEOS’). R Package Version 0.5-3. https://CRAN.R-project.org/package=rgeosraster.

[50] R Core Team (2020). R: A Language and Environment for Statistical Computing.

[51] Knight C.W., Bailey D.W., Faulkner D., Schafer D.W. Intake and grazing activity of mature range cows on Arizona rangelands. Proceedings of the Western Section American Society of Animal Science.

[52] Sprinkle J.E., Sagers J.K., Hall J.B., Ellison M.J., Yelich J.V., Brennan J.R., Taylor J.B., Lamb J.B. (2019). Grazing behavior and production for cattle on differing late-season rangeland grazing systems with or without protein supplementation. Transl. Anim. Sci..

[53] Earley A., Sowell B., Bowman J. (1999). Liquid supplementation of grazing cows and calves. Anim. Feed. Sci. Technol..

[54] Sowell B.F., Bowman J.G.P., Grings E.E., MacNeil M.D. (2003). Liquid supplement and forage intake by range beef cows. J. Anim. Sci..

[55] Wyffels S.A., Dafoe J.M., Parsons C.T., Boss D.L., DelCurto T., Bowman J.G.P. (2020). The influence of age and environmental conditions on supplement intake by beef cattle winter grazing northern mixed-grass rangelands. J. Anim. Sci..

[56] Walker R.S., Martin R.M., Gentry G.T. (2015). Impact of cow size on dry matter intake, residual feed intake, metabolic response, and cow performance. J. Anim. Sci..

